# Exploring the Impact of a Remote Monitoring System for Palliative and End-of-Life Care (CARE-PAC): Mixed Methods Feasibility Study

**DOI:** 10.2196/69394

**Published:** 2025-09-23

**Authors:** Roma Maguire, Lisa McCann, Claire Singleton, Paul Perkins, Ollie Minton, Nicola McCann, Emma Longford, Alistair Dermot McKeown, Kimberley Kavanagh, Morven Miller

**Affiliations:** 1 Department of Computer and Information Sciences University of Strathclyde Glasgow United Kingdom; 2 Department of Mathematics and Statistics University of Strathclyde Glasgow United Kingdom; 3 Sue Ryder, Leckhampton Court Hospice Cheltenham United Kingdom; 4 Sussex Cancer Centre University Hospitals Sussex NHS Foundation Trust Brighton United Kingdom; 5 NHS Lanarkshire Bothwell United Kingdom; 6 Wirral Hospice St John's Wirral United Kingdom; 7 Prince and Princess of Wales Hospice Glasgow United Kingdom

**Keywords:** PEOLC, palliative and end-of-life care, CARE-PAC, Care and Support System for Patients and Carers, remote monitoring, usability

## Abstract

**Background:**

In the United Kingdom, access to and the quality of palliative and end-of-life care (PEOLC) vary widely. In the final months of life, many patients face avoidable accident and emergency (A&E) visits and hospital admissions, driven by gaps in out-of-hours support and poorly coordinated care. This not only increases stress for patients and carers but also places avoidable strain and cost on the National Health Service (NHS). There is an urgent need for more compassionate, person-centered models that support people to remain at home, improve their quality of life (QoL), and reduce unnecessary use of acute services.

**Objective:**

This study aimed to explore the usability, user experiences, and impact of the digital dyadic remote monitoring Care and Support System for Patients and Carers (CARE-PAC) for patients in the last year of life, their informal carers, and health professionals involved in their care.

**Methods:**

Patients and informal carers were recruited to use CARE-PAC for up to 12 weeks. A mixed methods approach was used. Quantitative methods included the use of validated QoL scales and the System Usability Scale (SUS). Paired QoL and usability data were analyzed using the Wilcoxon (Pratt) signed-rank test, while unpaired usability data were analyzed using the Wilcoxon rank-sum test. Qualitative methods involved short catch-up calls, in-depth interviews, and focus groups conducted using topic guides informed by the domains of the Non-adoption, Abandonment, Scale-up, Spread, and Sustainability (NASSS) framework. Data were analyzed thematically.

**Results:**

CARE-PAC was implemented across 5 UK clinical sites with 26 participants (13 patient-carer dyads). No significant changes were observed in patients’ total QoL scores; however, significant improvements were seen in the “overall QoL” and “social” domains, alongside a significant decline in the “physical” domain. Carers showed no significant changes across total or domain-specific QoL scores. Usability was rated highly by patients (mean 87.9, SD 12.4) and carers (mean 94.7, SD 3.8), indicating an excellent user experience. Health care professionals (HCPs) reported lower usability scores (mean 63.6, SD 15.6), falling below average but above the threshold for poor usability. Thematic analysis of qualitative data gathered via catch-up calls (all patient-carer dyads), in-depth interviews (2 patients-2 carers), and 4 focus groups/1 interview (12 HCPs) identified 4 key themes: impact on care experiences, reflections and satisfaction, implementation challenges, and future directions.

**Conclusions:**

CARE-PAC is a usable, feasible, and acceptable remote monitoring and support system for patients in the last year of life, their carers, and HCPs. It enables real-time identification of needs and has shown positive impacts on the QoL of both patients and carers. These findings support the need for further research to evaluate its effectiveness at scale and explore pathways for wider implementation in PEOLC.

## Introduction

### Palliative and End-of-Life Care

The World Health Organization defines palliative care as an approach that improves the quality of life (QoL) of patients with life-limiting illnesses and their families by preventing and relieving suffering through early identification, assessment, and treatment of pain and other physical, psychosocial, and spiritual problems. It encompasses both palliative care, which can be provided alongside curative treatment, and end-of-life care, which is focused on the final phase of life [1].

There remains a significant unmet demand for palliative and end-of-life care (PEOLC) across many health systems. In the United Kingdom, over 600,000 people die each year, with 90% estimated to benefit from PEOLC—yet, access and quality vary widely. It is estimated that around 100,000 people who could benefit from palliative care die each year without receiving it [2]. This gap in provision has profound implications for both patients and their informal caregivers. For patients, inadequate access to timely and appropriate palliative care often results in poor symptom management, increased psychological distress, and diminished QoL during a highly vulnerable period [3]. Informal caregivers, typically family members or close friends, also experience substantial emotional and physical burdens. The lack of adequate support can lead to increased stress and anxiety, feelings of isolation, and difficulty coping during caregiving and in bereavement [4].

In addition to the human cost, there are substantial financial implications. The final year of life is widely recognized as one of the most resource-intensive periods for health services. In the United Kingdom, over 80% of health care spending in the final year of life is allocated to hospital care, while only 11% is directed toward primary and community health care and less than 4% to hospice care [5]. Although home-based interventions offer substantial savings to the health care system and improvements in patient and caregiver outcomes [6], inadequate support in the community often leaves patients and families with no alternative but to seek help through emergency routes, leading to avoidable accident and emergency (A&E) visits and hospital admissions [7]. These reactive and costly interventions are frequently a consequence of gaps in proactive, coordinated palliative care in the home or community setting [8].

### Can Technology Improve PEOLC Services?

Digital technologies have the potential to support, strengthen, and scale up palliative care worldwide [9]. The most common digital health interventions implemented in palliative care are videoconferencing, electronic health records, and phone calls, with the most typical usage of technologies being education, symptom management, decision-making, information provision, and management and communication [10]. eHealth apps have been shown to have promise in promoting equal, individualized care; facilitating accessibility and patient participation in palliative care settings; and promoting feelings of safety and security, while contributing to a more sustainable and efficient use of health care resources [11]. That said, digital technologies should be used to enhance—not replace—face-to-face interactions [12], ensuring that PEOLC services continue to deliver compassionate, person-centered care [13].

### Development of a Remote Monitoring and Support System for PEOLC

**Figure 1:**
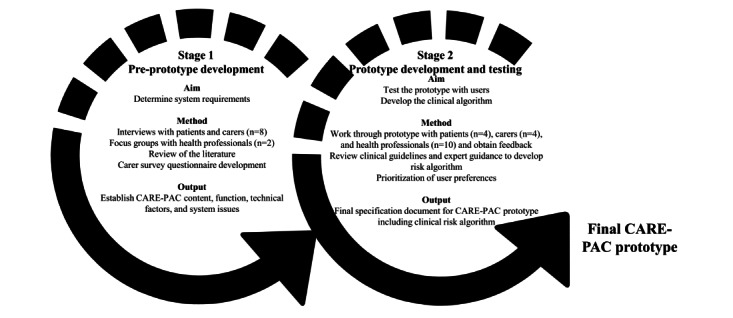
Co-design and development of the CARE-PAC app. CARE-PAC: Care and Support System for Patients and Carers.

**Figure 2:**
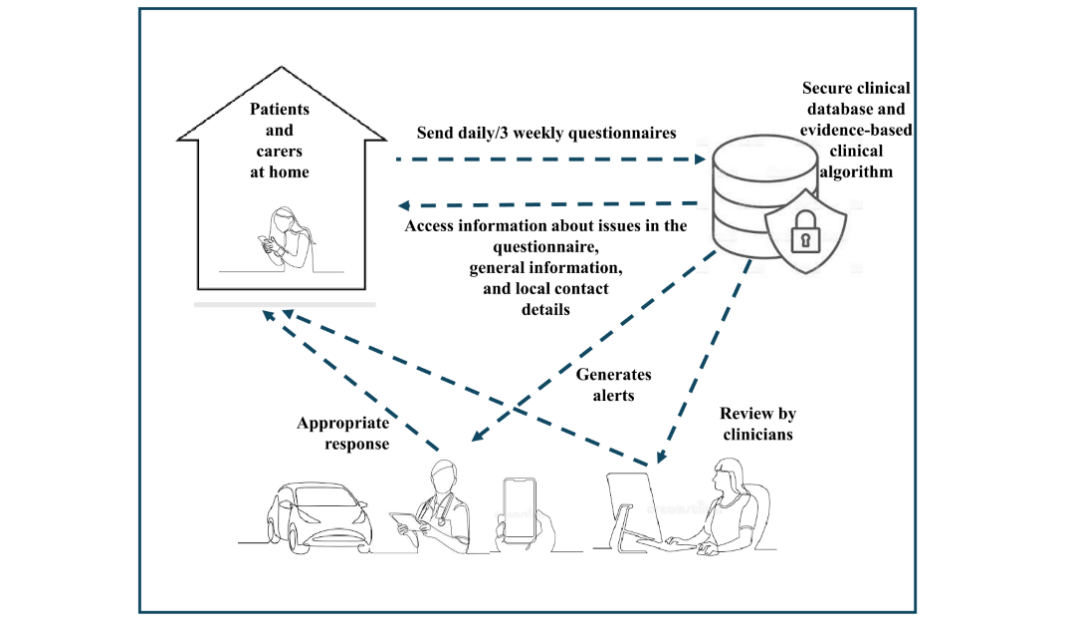
Final version of CARE-PAC: Care and Support System for Patients and Carers.

The Care and Support System for Patients and Carers (CARE-PAC) was co-designed over 2 stages (Figure 1) with patients, carers, and PEOLC health care professionals (HCPs). The result was a remote monitoring and supportive care system tailored to the specific needs of this population (Figure 2) that includes 3 connected components:

- Patient and Carer app: a mobile app for smartphones or tablets that enables users to (1) complete a brief daily symptom questionnaire, (2) submit a 3-weekly QoL and outcomes questionnaire, and access to an electronic library (e-library) featuring (1) web links to trusted websites aligned with questionnaire topics and (2) local service information (eg, helplines, support groups)
- Clinician dashboard: a secure, web-based platform accessible on any device that allows HCPs to (1) register and manage participants, (2) monitor daily symptom reports, and (3) receive and respond to real-time alerts
- Clinical algorithm: an embedded decision support tool that analyzes incoming data and flags concerning symptoms or issues, automatically generating alerts to HCPs via the clinician dashboard

A key strength of CARE-PAC is its dyadic nature through the inclusion of informal carers as active participants within the system, alongside patients themselves. This recognizes carers’ crucial role in supporting patients receiving PEOLC. By monitoring and supporting carers’ well-being, alongside that of patients’, CARE-PAC aims to help prevent carer crises that can lead to avoidable hospital admissions and to ensure more sustainable care at home.

### Aim of This Study

This study aimed to explore the usability, user experiences, and impact of CARE-PAC on the QoL, a digital dyadic remote monitoring and supportive care system for patients receiving PEOLC, their informal carers, and HCPs involved in their care.

## Methods

### Participants and Settings

Patient, informal carer, and HCP eligibility criteria for the study were set (Textboxes 1-3). The study was conducted in 5 clinical sites (3 hospices, 1 hospital, and 1 community setting) across Scotland and England. All participants were recruited from these 5 clinical sites. Patients and their informal carers were identified, approached, and recruited by HCPs involved in the study at these sites.

**Patient participation eligibility criteria.**

**Eligibility criteria:**

- Diagnosed with one or more of the following illnesses: advanced cancer (any diagnosis) and advanced chronic respiratory diseases, such as chronic obstructive pulmonary disease (COPD), pulmonary fibrosis, or bronchiectasis
- Aware of their advanced diagnosis
- Registering one or more indicators on the Supportive Palliative Care Indicators Tool (SPICT), including both general and disease-specific indicators, as completed by a member of the multidisciplinary team
- Receiving palliative care services
- Aged 18 years or over
- Have an informal carer who is also willing to participate in the study
- Able to provide informed consent
- Deemed to be physically, psychologically, and cognitively fit to participate in the study, as confirmed by a member of the multidisciplinary clinical team
- Able to read, write, and understand English and have a basic understanding of computer and audiovisual software (for data and outcome data collection purposes)
- If possible, have access to a PC, laptop, tablet, smartphone, or other equipment that can run the Care and Support System for Patients and Carers (CARE-PAC) system and support audiovisual communication software

**Informal carer participation eligibility criteria.**

**Eligibility criteria:**

- Identified by the patient (who meets the eligibility criteria listed in Textbox 1) as their key carer
- Aged 18 years or over
- Able to provide informed consent
- Able to read, write, and understand English and have a basic understanding of computer and audiovisual software (for data and outcome data collection purposes)
- If possible, have access to a PC, laptop, tablet, or smartphone or other equipment that can run the Care and Support System for Patients and Carers (CARE-PAC) system and support audiovisual communication software

**Health care professional participation eligibility criteria.**

**Eligibility criteria:**

- A member of the multidisciplinary team caring for patients in any of the diagnostic groups identified based on the eligibility criteria listed in Textbox 1
- Working in or across any clinical setting that provides care to patients in any of the diagnostic groups identified based on the eligibility criteria listed in Textbox 1
- Have access to a PC, laptop, or other equipment that can support audiovisual communication software and a have basic understanding of computer and audiovisual software

### Ethical Considerations

The study was approved by the North-West Preston Research Ethics Committee (reference 21/NW/0060) and sponsored by the University of Strathclyde. Research and development (R&D) approval was granted by each clinical site. The HCPs themselves provided informed consent to participate to the study’s research fellow. All participants (patients, informal carers, and HCPs) received both written and verbal information about the study, along with a hands-on demonstration of CARE-PAC, and were provided with an opportunity to ask questions before providing written informed consent.

To help address the digital divide—such as disparities in access to internet-enabled devices—participants without the necessary technology were offered smartphones with SIM cards on loan. To further support inclusion, in-person training was provided to all individuals (patients, carers, and HCPs), including guided demonstrations using a training version of the system to build user confidence and familiarity. Participants also received tailored user manuals (for patients, carers, and HCPs), which featured step-by-step instructions, annotated screenshots, frequently asked questions (FAQs), and contact details for further support. These resources aimed to promote equitable engagement with the digital intervention by addressing not only access but also skills, knowledge, and confidence.

### Day-to-Day Use of CARE-PAC

During the study, patients and their carers were invited to use CARE-PAC for up to 12 weeks, while HCPs at each clinical site participated throughout the study period.

#### Patients and Carers

Through the app, every day, patients and carers were prompted to complete a daily questionnaire. Patients completed the Integrated Palliative Care Outcome Scale (IPOS) [14], while carers completed the Digital Carer Daily Assessment (DiCDA), co-developed during stage 1 of CARE-PAC’s design. Responses were automatically uploaded in real time to a secure clinical database accessible to the health care team. The daily questionnaire could only be submitted once per day.

Every 3 weeks, patients and carers were prompted to complete outcome measures—specifically, a QoL questionnaire [15,16] and the System Usability Scale (SUS) [17]. These were accessed immediately following the daily questionnaire and remained available for 2 consecutive days before becoming inactive until the next 3-week cycle.

At any time, and as frequently as required, participants could access the e-library.

At the end of their involvement in the study, patients and carers were instructed to delete the app from their device.

#### HCPs

HCPs at each clinical site established local protocols for incorporating daily checks of the clinician website into their routine workflow. These daily reviews enabled them to:

- Access participants’ daily data reports to remotely monitor patients and carers, identify emerging symptom trends, and prioritize the clinical workload accordingly.
- View system-generated alerts, initiate appropriate clinical interventions, and document actions taken directly within the clinician website.

### Outcome Data Collection and Synthesis

Given the advanced stage of disease in the study population, an attrition rate of approximately 30% was anticipated [18]. To mitigate potential data loss due to disease progression or death, outcome data were collected on a regular 3-weekly schedule. This approach ensured that all participants got an opportunity to contribute their experiences and outcomes using mixed methods (see Table 1), irrespective of their duration in the study.

**Table 1:** Mixed data collection methods across the study population: patients, carers, and HCPs^a^.

Data collection method		Patient frequency	Carer frequency	HCP frequency
**Quantitative data collection**				
	QoL^b^ questionnaire	3-weekly	3-weekly	N/A^c^
	SUS^d^	3-weekly	3-weekly	Start and end
**Qualitative data collection**				
	Catch-up calls	3-weekly	3-weekly	N/A
	In-depth interviews	End of participation	End of participation	N/A
	Focus groups	N/A	N/A	End of study

^a^HCP: health care professional.

^b^QoL: quality of life.

^c^N/A: not applicable.

^d^SUS: System Usability Scale.

#### Quantitative Data Collection and Synthesis

##### Quality-of-Life Measurements

Patients’ QoL was measured using the 21-item McGill Quality of Life-Expanded (MQoL-E) [15], which assesses the following domains: physical, psychological, social support, existential/spiritual, cognition, health care, environment, and the feeling of being a burden. Carers’ QoL was measured using the 17-item Quality of Life in Life-Threatening Illness – Family Carer Version 3 (QOLLTI-F v3), a companion questionnaire to the MQoL-E that assesses 7 domains: environment, patient condition, caregiver’s own condition, caregiver’s outlook, QoL, relationships, and financial worries [16].

Both questionnaires were specific for this study’s patient and carer population and were deemed relevant, easy to understand, and reflective of important aspects of the QoL, making their completion meaningful and valuable [19]. The questionnaires typically take 10-20 minutes to complete, with the completion time often decreasing with increased familiarity. Local principal investigators approved the use of these QoL questionnaires for inclusion in the study.

##### System Usability Assessment

Usability was assessed using the SUS, a quick 10-item tool designed to measure users’ subjective perceptions of a system’s usability [17], proven to be both reliable and valid in assessing system learnability and usability [20]. A SUS score of 68 or higher is considered above average, while a SUS score above 85/below 50 indicates excellent/poor user experience, respectively [20]. An adjective rating scale has been shown to correlate extremely well with total SUS scores and can be used to help understand how a total score translates into a judgement of overall usability [21].

#### Qualitative Data Collection and Synthesis

##### Catch-Up Calls

All patients and carers received a catch-up phone call every 3 weeks from a research team member (author MM), lasting 5-10 minutes. Participants were asked a series of questions about their experiences with CARE-PAC. These conversations were audio-recorded using an encrypted digital device, and responses were extracted from the recordings, with participants’ views or verbatim quotes being documented in an anonymized, secure Microsoft Word document for each call.

##### Interviews and Focus Groups

At the end of participation, longer interviews were conducted with willing patients and carers, while all HCPs were invited to participate in focus groups. These interactions followed a topic guide based on the Non-adoption, Abandonment, Scale-up, Spread, and Sustainability (NASSS) framework [22], addressing topics such as perceptions of the technology, advantages and disadvantages of the system, and its impact/benefit. Both interviews and focus groups were audio-recorded using encrypted devices, transcribed verbatim, and reviewed for accuracy by a research team member prior to analysis.

### Data Analysis

Data from all 5 study sites were collated, and unknown participant IDs or duplicate entries were removed. If a question was answered multiple times on the same day, the most up-to-date response was taken and the others removed.

#### Quantitative Data Analysis

##### QoL Questionnaires and the System Usability Scale

For all questions the Wilcoxon (Pratt) signed-rank test was used as the statistical test of differences. This test was used as the differences analyzed were paired as responses at the first and the last completion related to the same participant. The Pratt specification of the Wilcoxon test was used as there were some differences of zero that were of importance. In a standard Wilcoxon signed-rank test, these zero difference responses would be discarded. For the HCPs’ SUS analysis, the Wilcoxon rank-sum test was used as baseline and endline responses were not paired.

##### Quality of Life

To examine differences in responses between the QoL questionnaire first and last completion, participants were included if they had at least 2 fully completed responses recorded on separate days. The difference in responses from first to last completion were examined at an individual question level, at a domain level using a domain score, and an overall QoL score level. To ensure a consistent QoL scale by domain, responses to some of the questions within each QoL questionnaire had to be transposed such that the new response was 10 minus the original response, which ensured that a response of 10 was the best QoL for each question. Within the patient QoL questionnaire, this related to 2 questions from the “physical” domain, 5 questions from the “thoughts and feelings” domain, and 1 question from the “social” domain. Within the carer QoL questionnaire, this related to 1 question in the “patient condition” domain, 1 question in the “your own condition” domain, 2 questions in the “relationships” domain, and 1 question in the “financial worries” domain.

##### System Usability Scale

Total SUS scores were calculated by first converting individual question responses to a consistent point system. This was done by subtracting 1 from the original score for odd-numbered questions and taking 5 minus the original score for even-numbered questions. Next, for each individual, their new responses to the 10 questions were summated, and the summed value was multiplied by 2.5. This gave a single total SUS score for each individual, which could take the value 0-100.

Mean (SD) values of overall and first and last SUS scores were calculated to examine differences in responses between the total SUS first and last completion score, and participants were included if they had at least 2 fully completed responses recorded on separate days.

#### Qualitative Data Analysis

Data from the catch-up calls, in-depth interviews, and focus groups were analyzed using thematic analysis, a method of identifying, analyzing, organizing, describing, and reporting themes found within a dataset [23]. NVivo software was used to analyze the data from in-depth interviews and focus groups. The initial coding framework was based on the interview/focus group topic guides, and data from the 2 groups (patients-carers and HCPs) were analyzed separately. Common overarching themes were identified in both the patient-carer and HCP data. However, there were instances where patients did not comment on specific subthemes highlighted by HCPs, as these were often related to the practicalities of conducting the research within clinical practice—an area outside the patients’ experiences.

Data analysis, coding, and thematic findings from the catch-up calls, in-depth interviews, and focus groups were conducted by one of the research team members (MM) using a systematic thematic analysis approach. To ensure the rigor and validity of the findings, the emerging themes and subthemes were regularly presented to the wider research team during collaborative meetings. These discussions provided an opportunity to reflect on the data, challenge interpretations, and ensure that the analysis remained grounded in the participants’ accounts. This collaborative review process facilitated a shared understanding of the data and supported the refinement of themes to ensure they accurately represented the findings. Through this iterative process, the research team concluded that thematic saturation had been reached, enhancing confidence in the robustness and credibility of the analysis.

## Results

### Overview

In total, 13 patient-carer dyads participated across the 5 clinical sites. Two participants, one patient, and one carer (unrelated) borrowed a study smartphone for the duration of their participation in the study.

#### Participants

The patient population consisted of 13 individuals (n=10, 76.9%, male; n=3, 23.1%, female) aged between 50 and 74 years. Most had a cancer diagnosis, including lung cancer (n=6, 46.2%), prostate cancer (n=3, 23.1%), pancreatic cancer (n=1, 7.7%), gastric cancer (1, 7.7%), and sarcoma (1, 7.7%), with 1 (7.7%) patient having a noncancer condition. The majority of carers were spouses (n=10, 76.9%), with others being siblings (n=2, 15.4%) or a parent (n=1, 7.7%).

The carer population comprised 13 individuals (n=11, 84.6% female; n=2, 15.4%, male), with ages ranging from 30 to 49 years.

The HCP group included 7 participants: 4 (57.1%) palliative care consultants and 3 (42.9%) research nurses or coordinators.

### Quantitative Results

#### Quality of Life

Table 2 shows the completion and inclusion rates for the QoL questionnaire among patients and carers.

All QoL questions were answered on every completed attempt. Participants with at least 2 fully completed responses on separate days were included in the analysis of change over time, which examined individual questions, domain-level scores, and overall QoL scores.

At an individual question level, there were significant differences in patients’ and carers’ responses to some questions within their QoL questionnaires (Table 3).

**Table 2:** Completion and inclusion rates for the QoLa questionnaire among patients and carers.

Group	Total participants, N	Completed at least once, n (%)	Completed only once, n (%)	Completed ≥2 times, n (%)	Highest number of completions, n; participants, n (%)	Included in first vs last analysis, n (%)
Patients	13	11 (84.6)	3 (23.1)	8 (61.5)	5; 2 (15.4)	8 (61.5)
Carers	13	9 (69.2)	2 (15.4)	7 (53.8)	5; 1 (7.7)	7 (53.8)

^a^QoL: quality of life.

**Table 3:** Questions from the patient and carer QoL^a^ questionnaires that had significantly different responses.

Questionnaire and domains		Question	Significance (%)	Direction of change
**MQoL-E^b^ questionnaire (patients)**				
	Overall QoL	Considering all parts of my life (eg, physical, emotional, social, spiritual, and financial) over the past 2 days (48 hours), the quality of my life was: Response: from “very bad” (0) to “excellent” (10)	5	Improved
	Physical	Over the past 2 days (48 hours), being physically unable to do the things I wanted was: Response: from “not a problem” (0) to “a huge problem” (10)	5	Declined
	Thoughts and feelings	Over the past 2 days (48 hours), I felt that the amount of control I had over my life was: Response: from “not a problem” (0) to “a huge problem” (10)	10	Declined
	Social	Over the past 2 days (48 hours) communication with the people I care about was: Response: from “difficult” (0) to “very easy” (10)	5	Improved
	Social	Over the past 2 days (48 hours), I felt my relationships with the people I care about were: Response: from “more distant than I would like” (0) to “very close” (10)	5	Improved
	Social	Over the past 2 days (48 hours), I felt supported: Response: from “not at all” (0) to “completely” (10)	5	Improved
**QOLLTI-F v3^c^ questionnaire (carers)**				
	Your outlook	Presently, I feel that my life has meaning: Response: from “very little meaning” (0) to “very much” (10)	10	Declined

^a^QoL: quality of life.

^b^MQoL-E: McGill Quality of Life-Expanded.

^c^QOLLTI-F v3: Quality of Life in Life-Threatening Illness – Family Carer Version 3.

Regarding domains within each of the QoL questionnaires, a domain score was calculated for each participant as the mean of responses to questions in the domain. The patient group had significantly different domain scores from first to last completion for the domains: improved “overall quality of life” domain score, improved “social” domain score, and decreased “physical” domain score. The carers had no significant differences in domain scores.

The total QoL score was calculated as the mean of the domain scores for each participant. Neither the patients nor carers had significantly different overall QoL scores from first to last response.

#### System Usability Scale

##### Completion Rates

The SUS questionnaire was completed at least once by 9 (69.2%) of 13 patients and 10 (76.9%) of 13 carers. Furthermore, 2 (15.4%) carers and 3 (23.1%) patients completed the questionnaire only once, while the maximum number of completions was 5, achieved by 2 (15.4%) carers and 2 (15.4%) patients. All SUS items were completed on every occasion by all participants.

Participants were included in the analysis of change over time if they completed at least 2 fully completed SUS questionnaires on separate days. This reduced the sample size to 6 patients and 8 carers.

The SUS questionnaire was also administered to HCPs at baseline and endline. In total, 23 HCPs completed the baseline questionnaire, although 1 (4.3%) HCP omitted 5 items (questions 3, 5, 6, 8, and 9). The endline questionnaire was fully completed by 18 (78.3%) HCPs.

##### Summary of SUS Scores

Table 4 shows a summary of SUS completion and scores across participant groups.

**Table 4:** Summary of SUS^a^ completion and scores across participant groups.

Group	Sample size, n/N (%)	SUS completion range	SUS score, mean (SD)	Highest number of completions, n; participants, n (%)	Significant change over time
Patients	9/13 (69.2)	1-5	87.9 (12.4)	5; 2 (15.4)	Yes (*P*<.10)
Carers	10/13 (76.9)	1-5	94.7 (3.8)	5; 2 (15.4)	No
HCPs^b^	Baseline: 23 (78.3) Endline: 18 (78.3)	1 (baseline and endline)	63.6 (15.6)	N/A^c^	No (*P*=.77)

^a^SUS: System Usability Scale.

^b^HCP: health care professional.

^c^N/A: not applicable.

The combined first and last SUS scores for patients and carers indicated excellent usability of the CARE-PAC app. Based on the adjective rating scale associated with SUS scores, carers rated the app as extremely usable (mean 94.7, SD 3.8), and patients also rated it as highly usable (mean 87.9, SD 12.4).

For HCPs, the mean SUS score across baseline and endline responses was 63.6 (SD 15.6), slightly below the industry-average benchmark of 68. This fell between the SUS adjective ratings of “OK” and “good.” A comparison of baseline and endline scores using an independent Welch *t*-test showed no statistically significant change in HCP usability scores (*P*=.77).

### Qualitative Outcomes

#### Themes Identified

Overarching themes and subthemes from patient, carer, and HCP qualitative data (catch-up calls and longer interviews/focus groups) were identified (Table 5).

The overarching themes (impact on care, satisfaction and reflections, challenges in project implementation, and future directions) were common across both patients and carers, with a few exceptions when subthemes were only identified by HCPs. Therefore, the themes from all participants (patients, carers, and HCPs) were reported together. Quotes for each subtheme, including identifiers to denote the group to which each participant belonged, can be found in Multimedia Appendix 1.

**Table 5:** Themes and subthemes from patient, carer, and HCP^a^ qualitative data.

Overarching theme	Subthemes
Impact on care experiences	Proactive monitoring and early detection QoL^b^, empowerment, and sense of worth Increased sense of reassurance and security Usefulness of reputable information
Reflections and satisfaction	Personal reflections Perceived added value
Challenges in project implementation	Technical issues Project population Recruitment Resources, workload, and staffing
Future directions	Sustainability Suggestions for improvement

^a^HCP: health care professional.

^b^QoL: quality of life.

#### Impact on Care Experiences

This theme related to the added value that CARE-PAC had on experiences of care delivery and receipt. Subthemes identified were proactive monitoring and early intervention, impact on QoL and sense of worth, increased sense of reassurance and security, and usefulness of reputable information.

#### Proactive Monitoring and Early Intervention

Patients, carers, and HCPs agreed that CARE-PAC enables faster, more effective symptom sharing and earlier intervention. Patients felt more responsible for tracking and understanding their symptoms, while HCPs valued the ability to spot changes early, benefitting health care generally through reducing the need for more costly interventions to address symptoms at a more advanced stage.

#### Impact on QoL and Sense of Worth

Participation in the study was reported to positively impact patients’ and carers’ QoL and sense of worth. Using the CARE-PAC app helped them reflect on their well-being, feel more in control, and contribute meaningfully to future care, which was also recognized and valued by HCPs.

#### Increased Sense of Reassurance and Security

Patients, carers, and HCPs felt CARE-PAC provides a sense of security and peace of mind, reassuring users that someone was monitoring them and helping patients and carers feel less alone.

#### Usefulness of Reputable Information

The CARE-PAC e-library was valued by patients and carers as a convenient and trustworthy source of information, offering symptom-specific resources and local service contacts in one place. Although not all users were aware of it, those who accessed it found it helpful.

#### Reflections and Satisfaction

This theme captured participants’ reflections on the personal significance of CARE-PAC and its broader impact on the palliative care community, highlighting the sense of satisfaction derived from using it. Two subthemes were identified, personal reflections and perceived added value.

#### Personal Reflections

Participants consistently reported that CARE-PAC is easy to use, making it easy to integrate into daily routines and professional practice. Patients and carers expressed a sense of loss when the study ended, reflecting its value in their lives. Although all groups recognized the growing importance of technology in health care and saw CARE-PAC as a timely and necessary innovation, they also acknowledged potential drawbacks, such as digital exclusion, increased anxiety, and reminders of illness. Despite these concerns, patients, carers, and HCPs all valued their involvement in the study, with many expressing gratitude for the opportunity to contribute.

#### Perceived Added Value

Patients, carers, and HCPs valued CARE-PAC’s ability to flag clinically concerning symptoms early, enabling timely interventions. The inclusion of carers’ perspectives was seen as a major strength, offering insight into both the patient’s condition and the carer’s well-being. CARE-PAC also helped improve communication between patients and carers, occasionally prompting meaningful discussions. HCPs appreciated being able to track symptom trends over time, helping them better prepare for patient contact and prioritize care based on clinical needs.

#### Challenges in App Implementation

Although most challenges were identified by HCPs, patients also highlighted some difficulties. Four subthemes emerged: technical issues; project population; recruitment; and resources, workload, and staffing.

#### Technical Issues

HCPs found that some parts of the clinician website are not user friendly, and patients identified awkward sections within the daily questionnaire.

#### Study Population

The digital divide and lack of confidence in using technology were seen as barriers. HCPs noted that some individuals are generally less engaged with health care. Late referrals meant many patients were too unwell to participate by the time the study was introduced to them. The dyadic design (patient and carer) posed recruitment challenges, especially when a carer was unavailable or hesitant or due to discomfort with the term “carer.”

#### Recruitment

HCPs found the research-related recruitment process time-consuming. They also experienced frustration with low referrals from other teams and acknowledged a tendency to gatekeep by not encouraging unsure patients to participate.

#### Resources, Workload, and Staffing

Although participation and managing alerts were manageable, HCPs raised ongoing concerns about limited staffing, time, and resources to support project delivery.

#### Future Directions

Building on the positive experiences of CARE-PAC, participants suggested future directions focusing on 2 main themes, sustainability and system improvements.

#### Sustainability

All participant groups emphasized the need for appropriate resourcing to ensure the long-term sustainability of CARE-PAC. HCPs highlighted the potential for a “snowball effect,” where increased use demonstrates value and encourages further adoption. CARE-PAC’s applicability to a wider range of palliative care conditions was seen as key to broader use. Identifying the optimal time to introduce CARE-PAC—when patients and carers can meaningfully engage—was considered vital. HCPs also recommended embedding CARE-PAC into routine care to ease implementation by removing additional research-related tasks.

#### Suggestions for Improvements

Participants proposed greater personalization of CARE-PAC, such as customizing alert timings, notification methods, and report frequency. Patients and carers expressed a desire to add context to daily reports, track symptoms over time, and include relevant notes (eg, pain medication) to enhance understanding and reduce unnecessary follow-ups. A key improvement suggested was integration across all health care teams, as patients felt frustrated that not all their care providers could access the clinician platform.

## Discussion

### Principal Findings

CARE-PAC is a practical and impactful remote monitoring and support system that holds significant value for the evolving field of digital PEOLC. Patients, carers, and HCPs consistently reported positive experiences and offered meaningful recommendations to guide future implementation and long-term sustainability.

### Comparison With Prior Work

The CARE-PAC feasibility study contributes to the growing body of work on digital palliative care systems. Comparing this study to prior work in the field reveals several key similarities and areas of advancement.

#### Symptom Monitoring, Early Detection, and Timely Intervention

Digital monitoring systems in palliative care have been shown to support early detection of escalating symptoms, enabling prompt clinical intervention and improving the QoL and patient-reported outcomes [24,25]. Platforms such as MyPal and similar oncology-based tools have demonstrated benefits, including better access to clinicians, faster responses, and more efficient care, particularly in rural settings [26-28]. Although CARE-PAC shares these strengths, it also introduces a novel dyadic approach by including carers in the monitoring process. This allows for proactive identification of carer concerns and well-being, enabling health care teams to respond to issues that, if left unaddressed, could lead to crisis situations, such as carer burnout and unplanned and unnecessary hospital admissions.

#### Patient and Carer Engagement

Engaging people nearing the end of life in research remains a challenge—often due to HCPs’ hesitancy to initiate these conversations, based on assumptions about the vulnerability of patients and families and concerns about placing an additional burden on them [29,30]. Ethical and practical concerns, such as challenges in gaining consent, avoiding undue distress, and balancing care needs with research demands, have also been cited as barriers [31-33]. CARE-PAC implemented established existing strategies to enhance user engagement [34,35], including involving patients and carers in co-design and testing to ensure the digital intervention is meaningful, usable, and relevant. The overwhelmingly positive feedback from patients, carers, and HCPs involved in CARE-PAC challenges these assumptions, demonstrating that meaningful involvement at this stage of life is both feasible and ethically justifiable.

Feedback loops have previously been used as a means of encouraging engagement with a digital system [27,28]. CARE-PAC provides 2 forms of feedback: an overview of patient and carer daily reports over time, enabling remote monitoring by health professionals, and a clinical risk algorithm that generates real-time alerts when submitted reports indicate clinically concerning issues. Patients and carers reported feeling a heightened sense of security and reassurance knowing that CARE-PAC would alert health care teams to any clinically concerning reports. This perceived value is reflected in the high compliance rates for completing the daily questionnaires—72.9% for patients and 65.5% for carers—which surpassed the completion rates for outcome measures (QoL and SUS), which had no immediate relevance or impact on their care. Future iterations of CARE-PAC will introduce a third feedback loop, enabling patients and carers to view their own symptom reports over time. This added feature has the potential to further enhance user engagement by promoting reflection and self-awareness.

#### Usability and Feasibility

Although digital health systems offer significant promise for remote monitoring in palliative care, usability and feasibility remain critical as poor design can hinder user adherence. Challenges, such as intrusive physical monitoring, privacy concerns, and rigid symptom reporting, persist [36,37]. However, mobile phone-based systems have shown strong acceptability—particularly in rural settings—when supported by user-centered design and insights from user experience research [36,38]. CARE-PAC’s mixed methods approach—integrating quantitative data with qualitative feedback—provides a comprehensive view of the user experience. In contrast with other systems [37,39], improvements in usability scores over time suggest CARE-PAC becomes more intuitive and user friendly with continued use.

### Strengths and Limitations

A key challenge in measuring experiences and outcomes in PEOLC is that patients may report a positive care experience despite poor clinical outcomes [40]. This challenge was evident in the CARE-PAC patient population, where individuals—despite receiving timely interventions, effective symptom management, and support—often experienced ongoing health deterioration due to the progressive nature of their condition.

#### Strengths

##### Co-design With Stakeholders

Engaging people receiving PEOLC in research can be challenging due to concerns about burdening vulnerable individuals, symptom severity, and misconceptions about the value of such research. Additionally, limited research infrastructure and funding in palliative care often restrict opportunities for meaningful inquiry. However, many patients and carers are motivated to participate, seeing it as a way to improve care for others in the future. CARE-PAC embraced this willingness by actively involving patients, carers, and HCPs in the co-design of the system. This collaborative approach ensured that the final prototype was not only technically functional but also aligned with the lived realities, priorities, and needs of intended users.

By designing the evaluation to capture data at 3-week intervals, the study ensured that participants’ experiences were included even if they withdrew or died before the study’s end. This approach reflects a compassionate and pragmatic design that acknowledges the realities of PEOLC. During the feasibility study, patients and carers consistently expressed gratitude for the opportunity to participate—reinforcing that, even in the context of advanced illness, people value meaningful engagement in research and can have positive experiences doing so.

##### Multiple Perspectives in Evaluation

One of CARE-PAC’s distinctive strengths is its inclusion of multiple perspectives in both its design and evaluation. The dyadic model—incorporating feedback and inputs from both patients and their informal carers—is a unique and innovative feature. Informal carers play a vital yet often undersupported role in delivering PEOLC, and their involvement in CARE-PAC acknowledges the multidimensional nature of caregiving. Including carers allowed for the identification of both patient condition and carer well-being, enabling HCPs to better understand the overall situation and potentially intervene to prevent carer burnout. Although clinicians noted some limitations in addressing carer-specific alerts due to the lack of background information, they saw value in these insights and suggested triaging carer needs through existing support services. Patients and carers also appreciated the real-time responsiveness of CARE-PAC, reporting that symptom data entered into the system influenced the support and treatment they received. The study’s design/methods also ensured that outcome data were collected even in cases of attrition, maximizing the utility of participants’ contributions regardless of duration of involvement.

##### Multisite Implementation

CARE-PAC was implemented across 5 clinical sites in Scotland and England, encompassing a range of care settings, including hospices, hospitals, and community services. The consistency of positive experiences across these diverse settings suggests that CARE-PAC has potential for broader generalizability. Patients and carers, regardless of location or diagnosis, engaged with the system in meaningful ways and reported similar benefits, supporting its potential scalability.

#### Limitations

##### Sample Limitations

###### Small Sample Size and Limited Diagnoses

The study involved a small sample of 13 patients (12 of whom had a cancer diagnosis) and 13 carers. Such a limited cohort restricts the generalizability of the findings and prevents the identification of statistically significant trends. The predominance of cancer diagnoses also limits insight into the system’s use across the wider palliative care population, including those with nonmalignant conditions.

###### Gatekeeping and Selection Bias

HCPs acknowledged challenges in recruiting participants and admitted to a degree of gatekeeping during the recruitment process. As a result, those who enrolled in the study may not fully represent the broader palliative care population. Participants were also likely to be more comfortable with technology, which may have positively skewed findings related to usability and acceptance. More broadly, difficulties in recruitment may reflect a well-recognized challenge within UK palliative care: the tendency for conversations about terminal illness and end-of-life care to occur too late or not at all [41]. These delays can prevent timely identification of individuals who would benefit from supportive interventions, such as CARE-PAC.

###### Dyadic Requirement

The requirement for participants to enroll as a dyad (patient and carer) introduced further limitations. This criterion reduced the pool of eligible participants, particularly where patients lacked an identifiable carer or where the carer did not see themselves in that role. Additionally, the dyadic model posed a dependency: when one member of the pair withdrew from the project—on 2 occasions—it forced the other, who may have wished to continue, to also withdraw.

###### Health Professional Involvement

Only small clinical teams from each site were involved in the study, making it difficult to assess how the system would perform at scale within wider, more complex health care systems.

###### Duration of Participation

The 12-week participation period, although appropriate and respectful of the health trajectories of people receiving PEOLC, limited the ability to evaluate the system’s long-term usability, sustainability, and impact. This time frame was practical, given anticipated symptom progression and potential attrition, but feedback indicated that some participants would have preferred to continue using CARE-PAC beyond 12 weeks. Several participants expressed a sense of loss when the study ended. A more flexible, patient-led participation period could be considered in future studies to better reflect user preferences and provide richer longitudinal data.

##### Data Collection and Evaluation Gaps

###### Impact of Alerts and Clinical Response

Although HCPs were required to acknowledge and close alerts via the clinician interface, there was no evaluation of these alerts, the interventions they triggered, or the resulting clinical outcomes. In addition, although patients and carers anecdotally reported that alerts prompted responsive care, these accounts were not formally assessed. Future research should include a structured evaluation of alert management and its clinical impact.

###### Quality-of-Life Data

The QoL dataset collected was small, limiting the conclusions that can be drawn about CARE-PAC’s impact on the QoL. Without robust data, improvements can only be cautiously interpreted.

##### Future Implications

To build on the early findings of CARE-PAC and address identified limitations, future research should aim to recruit a larger and more diverse participant sample, including individuals with a broader range of life-limiting conditions to evaluate the impact of CARE-PAC on patient and carer outcomes and cost-effectiveness of the system. Allowing patients and carers to engage either jointly or independently, and removing fixed end dates to give participants greater control over the duration of system use, may enhance flexibility and real-world relevance. Expanding clinician involvement across a variety of professional disciplines and health care settings will also be essential for assessing scalability, usability, and integration within routine clinical practice. Additionally, a formal evaluation of the clinical interventions triggered by CARE-PAC alerts is needed to better understand their impact on care quality, safety, health care costs, and patient and carer outcomes.

A significant insight from this study relates to the tension between research-related tasks and sustainable clinical implementation. Integrating CARE-PAC into standard models of care—rather than embedding it within research-only contexts—will be essential to its long-term viability. The requirement for tasks such as repeated consent visits and the distribution of information sheets created additional burdens that may hinder uptake in routine settings. These findings reflect wider challenges in palliative care research, where balancing research protocols with time-pressured, resource-limited clinical environments is often difficult. It is also notable that although several pilot studies on digital monitoring in palliative care have been published, few have reported on longer-term implementation, suggesting ongoing barriers to sustained integration.

CARE-PAC advances existing work in this area by offering a unique combination of features: high compliance; real-time data sharing; integrated, tailored information provision; and early responses from health care teams. Importantly, it also incorporates informal carers not only in their support roles but also as individuals with their own needs and well-being considerations. Its focus on early detection, proactive clinical intervention, and crisis prevention signals a shift toward a more anticipatory and comprehensive model of palliative care. Moreover, subthemes identified by HCPs around resourcing and triage point to the practical realities of embedding remote monitoring within overstretched services. These insights will be vital in shaping future models of care delivery.

To support sustainable implementation at scale, future development of CARE-PAC should prioritize 2 key areas. First, the interface for HCPs should be refined to ensure it presents the most relevant information in a concise, accessible, and user-friendly format, reducing information overload and supporting rapid decision-making. Second, CARE-PAC should be fully interoperable with existing electronic health record systems, allowing information captured via CARE-PAC to be viewed and acted upon within the digital platforms already used by the multidisciplinary care team. These improvements will facilitate seamless integration into everyday clinical workflows and enhance CARE-PAC’s appeal to providers. Broader deployment across a range of care settings—including community, hospice, and hospital services—will also be important to test CARE-PAC’s adaptability and utility in diverse real-world contexts. Ultimately, digital innovations like CARE-PAC have the potential to significantly improve the experience of PEOLC by supporting proactive, person-centered, and coordinated care for both patients and the people close to them.

#### Conclusion

The co-design and feasibility testing of CARE-PAC demonstrates promising potential to enhance the experiences of patients receiving PEOLC and their informal carers. Reported benefits include an improved QoL, earlier detection of symptoms enabling timely interventions, increased reassurance, and better access to tailored information and support. These findings underscore the value of digital health solutions in addressing some of the complexities and uncertainties of PEOLC, while supporting a more proactive, person-centered approach.

Nevertheless, several implementation challenges must be addressed. Recruitment was difficult, given the frailty and declining health of many eligible participants, and future delivery will require dedicated resources to support ongoing use in clinical practice. As health and social care services face increasing pressure due to rising demand and limited capacity, digital innovations like CARE-PAC offer a timely and scalable opportunity to strengthen palliative care provision. With appropriate investment and integration into routine systems of care, such approaches could help ensure that patients and informal carers receive responsive, compassionate, and coordinated support as they navigate the final stages of life.

## Appendix

Multimedia Appendix 1 Participant quotes supporting qualitative themes.

## Abbreviations

A&E: accident and emergency
CARE-PAC: Care and Support System for Patients and Carers
e-library: electronic library
HCP: health care professional
MQoL-E: McGill Quality of Life-Expanded
NASSS: Non-adoption, Abandonment, Scale-up, Spread, and Sustainability
NHS: National Health Service
PEOLC: palliative and end-of-life care
QoL: quality of life
QOLLTI-F v3: Quality of Life in Life-Threatening Illness – Family Carer Version 3
SUS: System Usability Scale

## Data Availability

The datasets generated and analyzed during this study are available from the corresponding author upon reasonable request.
