# Supplementary material for: Exploring the Impact of a Remote Monitoring System for Palliative and End-of-Life Care (CARE-PAC): Mixed Methods Feasibility Study
Source: JMIR Form Res. 2025 Sep 23;9:e69394. doi: 10.2196/69394 (PMC12504897; doi:10.2196/69394)
Supplement: Multimedia Appendix 1 [file formative_v9i1e69394_app1.docx]

Participant Quotes Supporting Qualitative Themes.

| **Theme/sub-theme** | **Relates Quotes** |
| --- | --- |
|  |  |
| **Impact on care experiences** | |
| Proactive monitoring and early detection | ‘It’s good in as much as things can be flagged up almost immediately…my pain and shortness of breath has been flagged up, my palliative care nurse got notified and I’m getting treated for that better now than before.’ (patient 1, site 1)  ‘[I was using it to almost monitor myself against a range. So for instance, tiredness, I've been struggling with tiredness. So when I put a three in, in my mind it means ‘OK, I've done this and that's why I'm tired’](https://strath-my.sharepoint.com/personal/morven_miller_strath_ac_uk/Documents/Transcribed%20Files/Cheltenham%20patient%20%233%20longer%20interview%201.MP3).’ (patient 3, site 4)  ‘[If you can actually have a fairly large percentage of the population that can benefit from CARE-PAC, yeah, that strikes me as a very cost effective way of actually being able to do wide scale sort of monitoring of them.](https://strath-my.sharepoint.com/personal/morven_miller_strath_ac_uk/Documents/Transcribed%20Files/Your%20Recording%201.wav)’ (carer 1, site 4)  ‘I [think it's got potential. I think it might need a bit of tweaking to make it more useful but I think the early monitoring and picking up symptoms is helpful](https://strath-my.sharepoint.com/personal/morven_miller_strath_ac_uk/Documents/Transcribed%20Files/Cheltenham%20HCP%20focus%20group%2005.12.23%202.MP3).’ (health professional 2, site 3) |
| QoL, empowerment and sense of worth | ‘The app is great, it does force you to stop and think about the situation, about how I’m feeling, which is a good thing, because it’s making me stop and actually think about what’s going on rather than ploughing through every day and not really taking a step back and breathing a little bit.’ (carer 3, site 4)  ‘[I think she liked the control, it gave her that bit of control, didn't it? I think they enjoyed the process of being involved in research as well as using the tool. They felt that feeling of doing something to give back and that they were useful. For them to do something that felt important was really useful. And so the process of being involved in the trial was as valuable for them as the trial itself.](https://strath-my.sharepoint.com/personal/morven_miller_strath_ac_uk/Documents/Transcribed%20Files/NHSL%20clinical%20longer%20end%20of%20study%20focus%20group.MP3)’ (health professional 1, site 3) |
| Increased sense of reassurance and security | ‘It’s almost as if you have another port of call, if something does go wrong and you can’t get in touch with the nurses, and he is going downhill now, and there’s been a couple of times that I’ve said ‘put it on your app’ and they (the nurses) have picked it up the following day and been on the phone and then come out.’ (carer 1, site 1)    ‘[I think the patients appreciated the support, that extra bit of support that the app gave them](https://strath-my.sharepoint.com/personal/morven_miller_strath_ac_uk/Documents/Transcribed%20Files/Cheltenham%20HCP%20focus%20group%2005.12.23%202.MP3), [this expression that there was an extra layer of care in a way that they wouldn't get missed. That was the positive feedback that I had from them.](https://strath-my.sharepoint.com/personal/morven_miller_strath_ac_uk/Documents/Transcribed%20Files/Cheltenham%20HCP%20focus%20group%2005.12.23%202.MP3)’ (health professional 2, site 4) |
| Usefulness of reputable information | ‘the times that I did look up the information I thought it was spot on. It did seem to be able to direct you to the places that you needed to go to.’ (patient 1, site 4)  ‘She (the carer) actually found great support from the links. She said ‘just that I can press a button and I don’t have to go into google and open up a can of worms. I can get into a good website here and get a wee look at things to help me’. She found that really reassuring.’ (health professional 2, site 1) |
|  |  |
| **Reflections and satisfaction** | |
| Personal reflections | [‘We [the carer and her husband] carried on using it at first when we should have stopped. It became a habit. It’s only been this last week we stopped using it.’ (carer 1, site 3)](https://strath-my.sharepoint.com/personal/morven_miller_strath_ac_uk/Documents/Transcribed Files/Your Recording 1.wav)  ‘[Everybody knows that the NHS can't continue the way it is at the moment because it it's just overwhelmed, and we also know there isn't, unlimited money to throw at it. So I think it would be a general perception that technology has got to play a bigger part. And therefore apps like this will come into their own as that perception is gaining common ground across the whole population.](https://strath-my.sharepoint.com/personal/morven_miller_strath_ac_uk/Documents/Transcribed%20Files/Cheltenham%20patient%20%233%20longer%20interview%201.MP3)’ (patient 1, site 4)  ‘As a principle, I think it’s (technology) the future. You know, I think patients taking control of their, their wellbeing and being able report things in real time and be monitored by a healthcare professional is the future. [You can see how things can be improved just by doing this project](https://strath-my.sharepoint.com/personal/morven_miller_strath_ac_uk/Documents/Transcribed%20Files/Brighton%20HCP%20end%20of%20study%20focus%20group%2007.12.23.MP3).’ (health professional 2, site 5)  ‘[And there were a few patients that I felt they had the technical literacy, but no, when we explored it further, they said ‘that’s just beyond me’.](https://strath-my.sharepoint.com/personal/morven_miller_strath_ac_uk/Documents/Transcribed%20Files/PPWH%20longer%20end%20of%20study%20focus%20group%2013.12.23.MP3) … [It was surprising how many people actually said IT was the issue. You know, they just didn't want to use another bit of technology and maybe they were sick of it. But you know, there was a few who just felt it wasn’t for them.](https://strath-my.sharepoint.com/personal/morven_miller_strath_ac_uk/Documents/Transcribed%20Files/PPWH%20longer%20end%20of%20study%20focus%20group%2013.12.23.MP3)’ (health professional 2, site 2)  ‘[She just decided it wasn’t for her. She liked the thought of it but the reality of it just made her think of all the things that were wrong with her.’ (health professional 3, site 1)](https://strath-my.sharepoint.com/personal/morven_miller_strath_ac_uk/Documents/Transcribed%20Files/NHSL%20clinical%20longer%20end%20of%20study%20focus%20group.MP3)  ‘[My overall emotion really is one of gratitude for being able to participate in the project.](https://strath-my.sharepoint.com/personal/morven_miller_strath_ac_uk/Documents/Transcribed%20Files/Cheltenham%20HCP%20focus%20group%2005.12.23%202.MP3)’ (health professional 1, site 4) |
| Perceived added value | ‘For instance, the clot in my leg…now if that hadn’t been picked up, I could have carried on for however long and by the time I went for a scan it would have been 10 times worse. And instead of just being on medication, I might have needed more intrusive treatment… so stopping that was a good thing.’ [patient 3, site 4]  ‘[One of the real benefits, I think of the app is the fact that it's looking at it from 2 perspectives. You know it's looking at it from the patient’s point of view as well as the carer’s. And often it's the carer who suffers, you know, probably as much if not more than the patient. But I think to be able to triangulate between the two that, you know, is really important… And that's a real value because too often, if I go into see a consultant or specialist nurse or any anybody else, they tend to very much focus on the patient… so having that way of, you know, being able to support the carer as well as the patient, I think is a real bonus of the app.](https://strath-my.sharepoint.com/personal/morven_miller_strath_ac_uk/Documents/Transcribed%20Files/Your%20Recording%201.wav)’ (patient 1, site 4)  ‘It definitely has benefits for both of us, it means he say what he wants on his (app) and I can say what I want on mine…so you’re not thinking, ‘oh I can’t say that because he might not want me to say that’, so it’s confidential, which is good as well.’ (carer 1, site 1)  ‘[And the mother said that it helped their relationship much more because they were asking each other what they were putting and how they might answer questions. And the mother said it's really opened up communication with her daughter, which is a nice thing and unexpected perhaps, but a nice thing for them so that was good.’](https://strath-my.sharepoint.com/personal/morven_miller_strath_ac_uk/Documents/Transcribed%20Files/Brighton%20HCP%20end%20of%20study%20focus%20group%2007.12.23.MP3) (health professional 3, site 5)  ‘[I used it to sort of track them, you know, because we were so busy. And I was having to look at my time and think about who were the people that I needed to see the most. And I could look at their responses and think, well, for him things are pretty steady at the moment. So that was quite useful because I could use it as a tool for checking](https://strath-my.sharepoint.com/personal/morven_miller_strath_ac_uk/Documents/Transcribed%20Files/Wirral%20HCP%20end%20of%20study%20longer%20interview%2019.12.2023.MP3).’ (health professional 1, site 3) |
|  |  |
| **Challenges in project implementation** | |
| Technical issues | ‘[it would be good if you could click on multiple alerts. So what I was doing is writing some text and copying and pasting that into each alert. But if I'd spoken to that patient that day and we came up with a plan that would be nice to just be able to knock out all those 10 alerts with the one sentence](https://strath-my.sharepoint.com/personal/morven_miller_strath_ac_uk/Documents/Transcribed%20Files/Brighton%20HCP%20end%20of%20study%20focus%20group%2007.12.23.MP3).’ (health professional 1, site 2)  ‘Some bits (of the app) are slightly repetitive…the bit where you do your 3 more symptoms, you have to put ‘not applicable’ three times, that’s a bit annoying.’ (patient 4, site 4) |
| Project population | ‘There were patients that I'd think ‘oh they'd be really good for this and be really engaged’. One lady who springs to mind in particular would have been perfect for it. And I spoke to her, and she was really positive, and she went home with the information. And then she came back and just said, ‘No, it's not my thing’. And you know she couldn't express why it wasn’t for her. And I think for her it was an extra thing that she didn't need at that time, which was a shame, because she would have been really good for it (the project).’ (health professional 1, site 3)  ‘[Probably years ago we would have had more appropriate patients because we saw people for a longer period of time. That's been a definite change…We take people when they're much more symptomatic and much nearer end of life now than ever.](https://strath-my.sharepoint.com/personal/morven_miller_strath_ac_uk/Documents/Transcribed%20Files/NHSL%20clinical%20longer%20end%20of%20study%20focus%20group.MP3)’ (health professional 3, site 1)  ‘[I don't know whether that (recruiting a dyad) put the CNSs off by making it more complicated. I think if it had just been a solo offer to the patients, maybe the perception would have been ‘it's less work’.](https://strath-my.sharepoint.com/personal/morven_miller_strath_ac_uk/Documents/Transcribed%20Files/Brighton%20HCP%20end%20of%20study%20focus%20group%2007.12.23.MP3) [I’m sure if we’d said to the specialist nurses ‘any one patient, send them to us’, we'd have got loads whereas it's when they're scratching their heads about whether they've got a carer or not. It just closed it off a bit, didn’t it?](https://strath-my.sharepoint.com/personal/morven_miller_strath_ac_uk/Documents/Transcribed%20Files/Brighton%20HCP%20end%20of%20study%20focus%20group%2007.12.23.MP3)’ (health professional 1, site 5)  ‘One thing I didn’t like was being called a ‘carer’, I see myself as her husband, I always have and I always will. I don’t like being referred to as a ‘carer’ or to be answering questions as a ‘carer’.’ (carer 1, site 2) |
| Recruitment | ‘[Once you’d decided that you’d have the chat with them, that took quite a chunk out your day. You know the discussions, the consent, the setting it up and all that. That was quite a lot of time.’](https://strath-my.sharepoint.com/personal/morven_miller_strath_ac_uk/Documents/Transcribed%20Files/NHSL%20clinical%20longer%20end%20of%20study%20focus%20group.MP3) (health professional 2, site 1)  ‘At [the start, at the very start, there were lots of them [CNSs] saying, ‘I can think of a patient now’ or ‘I've got someone in mind now’ and then when it actually came to doing it, it didn't come off.](https://strath-my.sharepoint.com/personal/morven_miller_strath_ac_uk/Documents/Transcribed%20Files/Brighton%20HCP%20end%20of%20study%20focus%20group%2007.12.23.MP3)’ (health professional 2, site 5)  ‘[We’re protecting the patient. I think that's probably what happened with me with definitely one of my patients because if I pushed it a wee bit further, I probably could have got them to do it (participate in the project). But things changed and they were hospitalised. And it was too much, I felt it was too much for them to participate and for me to push it.](https://strath-my.sharepoint.com/personal/morven_miller_strath_ac_uk/Documents/Transcribed%20Files/PPWH%20longer%20end%20of%20study%20focus%20group%2013.12.23.MP3)’ (health professional 2, site 2) |
| Resources, workload and staffing | ‘[Ideally we'd also have worked with the Community Specialist Palliative Care Nurses. But I think the feeling was in our setting that they were just so stretched that that they wouldn't be able to take this on.’ (health professional 1, site 4)](https://strath-my.sharepoint.com/personal/morven_miller_strath_ac_uk/Documents/Transcribed Files/Cheltenham HCP focus group 05.12.23 2.MP3)  ‘[I think that additional help for checking the alerts, so a dedicated person who was checking the alerts, would help. But that doesn't help with the actual time taken for the nurses actually following up on the alerts which I would say is the more time-consuming part of it.](https://strath-my.sharepoint.com/personal/morven_miller_strath_ac_uk/Documents/Transcribed%20Files/Cheltenham%20HCP%20focus%20group%2005.12.23%202.MP3)’ (health professional 1, site 1) |
|  |  |
| **Future directions** | |
| Sustainability | ‘[I think there's two things isn't there. Whether you need people specifically to train the patients and families or whether you are talking about digital team on the receiving end of the of these things. It should be possible to have it part of the healthcare professional’s job to have this and it should really make them more efficient. But I don't know whether you're going to need extra staff to training up patients and families to use it.](https://strath-my.sharepoint.com/personal/morven_miller_strath_ac_uk/Documents/Transcribed%20Files/Cheltenham%20HCP%20focus%20group%2005.12.23%202.MP3)’ (health professional 2, site 4)  ‘[I think it's a culture, a cultural thing. If we were all using something it becomes part of the culture, and we get to the stage that people would be more open to it.](https://strath-my.sharepoint.com/personal/morven_miller_strath_ac_uk/Documents/Transcribed%20Files/Brighton%20HCP%20end%20of%20study%20focus%20group%2007.12.23.MP3)’ (health professional 1, site 5)  ‘I [think it could quite easily be transferred to other long term illnesses quite easily but I think you would have to be fairly clear to people that, you know, this was aimed at people with cancer or people with MS or ME. But that's easy to do I think with the technology.](https://strath-my.sharepoint.com/personal/morven_miller_strath_ac_uk/Documents/Transcribed%20Files/Your%20Recording%201.wav)’ (patient 1, site 4)  ‘[If you established it either at the very start of someone being involved in community services or before they get to that end stage. So then people who are in clinic and used to that process being there and then, you're quite right, when things are changing, moving towards end of life, it's already an established process.’](https://strath-my.sharepoint.com/personal/morven_miller_strath_ac_uk/Documents/Transcribed%20Files/PPWH%20longer%20end%20of%20study%20focus%20group%2013.12.23.MP3) (health professional 1, site 2)  [And as our services are increasingly stretched, looking at different ways that we can manage people is really important. So I absolutely think that there is a place for it. I just think we just need to find a way of integrating that into the normal workload to make it just part of day-to-day work.](https://strath-my.sharepoint.com/personal/morven_miller_strath_ac_uk/Documents/Transcribed Files/Wirral HCP end of study longer interview 19.12.2023.MP3)  [[So I think if it wasn't part of a trial, that would probably be a bit more straightforward because you wouldn't need to have all of that extra paperwork, you'd just say this is what it is, do you want to use it? And so I think that would be a bit more straightforward, but I think all that information (related to the research aspect) was a bit much for some people.](https://strath-my.sharepoint.com/personal/morven_miller_strath_ac_uk/Documents/Transcribed Files/Wirral HCP end of study longer interview 19.12.2023.MP3)](https://strath-my.sharepoint.com/personal/morven_miller_strath_ac_uk/Documents/Transcribed%20Files/NHSL%20clinical%20longer%20end%20of%20study%20focus%20group.MP3)[’ (health professional 1, site 3)](https://strath-my.sharepoint.com/personal/morven_miller_strath_ac_uk/Documents/Transcribed Files/Wirral HCP end of study longer interview 19.12.2023.MP3) |
| Suggestions for improvement | ‘[How about that you could individualise it for patients? So there are some patients where you know that they're going to be scoring high and actually that you don't need to do anything about that because they don't want to change anything. Or maybe there's nothing you can do and that's just how they are. Then you wouldn't want alerts from that patient, but maybe you would want alerts from someone where you know they're doing pretty well and things might change, or you worry that they might change suddenly.](https://strath-my.sharepoint.com/personal/morven_miller_strath_ac_uk/Documents/Transcribed%20Files/Brighton%20HCP%20end%20of%20study%20focus%20group%2007.12.23.MP3)’ (health professional 1, site 4)  ‘[If I could go back and see the information that I’d already entered into it, but because I can't see that I had to manually track that and that becomes that becomes a bit of a mission](https://strath-my.sharepoint.com/personal/morven_miller_strath_ac_uk/Documents/Transcribed%20Files/Cheltenham%20patient%20%233%20longer%20interview%201.MP3)…[I would like the app to change, to be bit more pertinent to, to give me information more than me just feed it](https://strath-my.sharepoint.com/personal/morven_miller_strath_ac_uk/Documents/Transcribed%20Files/Cheltenham%20patient%20%233%20longer%20interview%201.MP3) …[because if you could put context to it and a rating system, and you can actually show individual trends, I think you would get a huge difference in your adoption population and rate, especially if you can show them the value of their own analysis.](https://strath-my.sharepoint.com/personal/morven_miller_strath_ac_uk/Documents/Transcribed%20Files/Cheltenham%20patient%20%233%20longer%20interview%201.MP3)’ (patient 3, site 4)  ‘If only the app was available to the GP and the oncologist and CNS at the hospital as well as my palliative care nurses, that would be really helpful.’ (patient 4, site 1)  ‘[I think it needs to be linked in, completed integrated. You shouldn't even have to put any details in at all because it's just there. And this would be just plug in.’ (health professional 1, site 5)](https://strath-my.sharepoint.com/personal/morven_miller_strath_ac_uk/Documents/Transcribed%20Files/Brighton%20HCP%20end%20of%20study%20focus%20group%2007.12.23.MP3) |
